# Encapsulation of mRNA in Therapeutics Like Lipid Nanoparticles Probed by Deep-UV Resonance Raman Spectroscopy

**DOI:** 10.1021/acs.analchem.5c04246

**Published:** 2026-01-15

**Authors:** Sila Jin, Sergei V. Reverdatto, Vladimir V. Ermolenkov, Alexander Shekhtman, Young Mee Jung, Igor K. Lednev

**Affiliations:** † Department of Chemistry, 1084 University at Albany, SUNY, 1400 Washington Avenue, Albany, New York 12222, United States; ‡ Kangwon Radiation Convergence Research Support Center, 34962 Kangwon National University, Chuncheon 24341, Korea; § The RNA Institute, College of Arts and Science, University at Albany, SUNY, 1400 Washington Avenue, Albany, New York 12222, United States; ∥ Department of Chemistry, Institute for Molecular Science and Fusion Technology, Kangwon National University, Chuncheon 24341, Korea; ⊥ Center for Biophotonic Technology and Artificial Intelligence (CeBAI), University at Albany, SUNY, 1400 Washington Avenue, Albany, New York 12222, United States

## Abstract

Messenger RNA (mRNA) therapeutics have emerged as a powerful platform for treating a wide range of diseases, but their clinical success depends on overcoming the issues of instability and delivery. mRNAs are typically unstable and require encapsulation in lipid nanoparticles (LNPs) for efficient delivery. This study quantifies free and encapsulated mRNAs within LNPs in a model vaccine using deep-UV resonance Raman (DUVRR) spectroscopy. DUVRR spectroscopy with excitation at 266 nm, matching the maximum UV absorption of mRNA, enables spectral differentiation of mRNA based on its degree of encapsulation—from fully encapsulated to partially exposed to completely free. Raman spectra were acquired from the samples with progressively decreasing lipid concentrations while maintaining a fixed mRNA concentration; vibrational modes associated with mRNA–lipid interactions were identified. Principal component analysis (PCA) revealed spectral variations linked to the lipid presence, especially between 1270 and 1800 cm^–1^. Two-trace two-dimensional correlation spectroscopy (2T2D-COS) was employed to extract the 1322 cm^–1^ band as a quantitative spectroscopic indicator of the lipid–mRNA interaction. This is the first use of 2T2D-COS for quantitative analysis of complex (bio)­chemical systems. The work highlights the unsurpassed potential of DUVRR spectroscopy to analyze mRNAs within LNPs.

## Graphical abstract

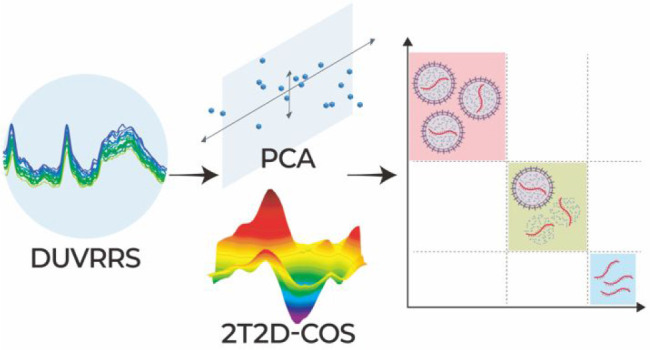

## Introduction

Since Karikó et al.^1^ first reported the potential for mRNA-based vaccine development in 2005, research into mRNA vaccines has steadily progressed. The COVID-19 pandemic marked the first case of mRNA vaccines being commercialized, positioning them as a next-generation vaccine technology.

Both unmodified and N1-methylpseudouridine-modified mRNAs are highly unstable, so the mRNAs must be encapsulated in lipid nanoparticles (LNPs) for storage, transport, and delivery. LNPs typically comprise of PEGylated lipids, cholesterol, phospholipids, ionizable lipids, and cationic lipids.^2^ In this study, for simplicity of terms, all components of the LNP formulation are referred to as lipids. Each type of lipid plays a specific role in the delivery of mRNA. For example, PEGylated lipids in LNPs serve as steric stabilizers, reducing nonspecific binding to proteins, while cholesterol and phospholipids, such as 1,2-distearoyl-*sn*-glycero-3-phosphocholine (DSPC), assist in particle stability, bioavailability, and biodistribution. Most LNP formulations rely on ionizable lipids, such as SM-102, to facilitate the endosomal escape of LNPs from the cytosol.^3,4^ The interaction between the negatively charged phosphate groups of mRNA and the ionizable lipids is crucial for encapsulation.^2^ This interaction can potentially be detected by Raman spectroscopy and can affect Raman bands, such as those associated with the ribose moiety of RNA, since the ribose is positioned closer to the phosphate group than the nucleobase.

The mixture of mRNA and lipids is naturally assembled into LNPs through electrostatic interactions, and the LNPs facilitate the delivery of mRNA across the cell membrane into the cytosol. Determining how much mRNA is encapsulated versus how much remains LNP-free is a critical factor in evaluating the efficiency of mRNA vaccine production.^5^ Raman spectroscopy offers the advantage of obtaining the intrinsic spectrum of analytes in aqueous solutions, as it is not affected by water interference and allows us to perform nondestructive sample analysis. In conventional Raman spectroscopy, autofluorescence from biomolecules can pose a significant challenge. However, by using deep-UV excitation (200–280 nm), fluorescence is largely avoided, facilitating a high signal-to-noise ratio in Raman spectra, which is beneficial for analyzing biomolecules and complex compounds. Particularly, when the excitation source is chosen in the wavelength range of an electronic transition of an individual chromophore group, resonance effects can significantly enhance the Raman signal, providing a highly specific Raman spectrum. Previous work by Almehmadi et al.^6^ demonstrated the significant potential of deep-UV resonance Raman (DUVRR) spectroscopy for assessing the stability of mRNA vaccines encapsulated within lipid nanoparticles (LNPs).

In the present study, rather than focusing on stability, we investigate the encapsulation states of mRNA within LNPs and their intermolecular interactions to evaluate the encapsulation efficiency of mRNA vaccines. To create an environment in which both encapsulated and unencapsulated mRNA coexist as shown in Figure 1, we systematically decreased the lipid content during encapsulation. This approach enabled us to quantitatively assess the distribution between the two states. Additionally, by using an excitation source at 266 nm, which corresponds to the maximum UV absorption wavelength of mRNA, we induced the resonance enhancement of the Raman signal from the mRNA, as previously demonstrated by Almehmadi et al.^6^ To resolve overlapping bands and analyze subtle spectral changes related to lipid–mRNA interactions, we integrated DUVRR spectroscopy with two-dimensional correlation spectroscopy (2D-COS), incorporating principal component analysis (PCA) and two-trace two-dimensional correlation spectroscopy (2T2D-COS). This combined analytical approach enhances spectral interpretation of the complex mixtures containing overlapping vibrational bands from lipid-bound and free mRNA and enables the extraction of quantitative correlation markers that characterize the encapsulation process.^7–9^

**Figure 1:**
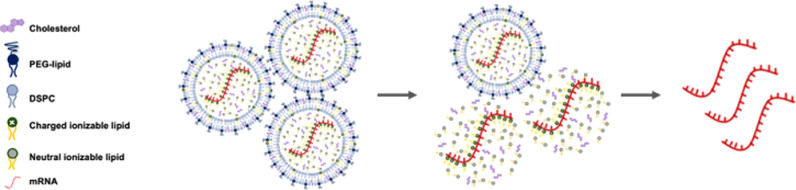
Illustration of mRNA–lipid nanoparticle (LNP) formulations with a decreasing lipid content. From left to right: fully encapsulated mRNA, partially encapsulated mRNA, and free mRNA without lipids.

## Experimental Section

### Materials

Adenosine monophosphate (AMP) was purchased from Sigma-Aldrich. Plasmid pTK305 encoding firefly luciferase was obtained from Addgene (#66812). N1-methylpseudouridine-triphosphate was purchased from TriLink Biotechnologies. Ascl restriction enzyme and RNase-free TE buffer were obtained from New England Biolabs and Thermo Fisher Scientific, respectively. The MEGAscript T7 Transcription Kit was obtained from Ambion for in vitro transcription. LiCl and EDTA for RNA purification were purchased from Sigma-Aldrich. Lipofectamine 2000, used for mRNA encapsulation, was purchased from Thermo Fisher Scientific. SH buffer was prepared using NaCl and HEPES (Sigma-Aldrich), and sodium sulfate (Na_2_SO_4_, Acros Organics) was used as an internal standard. All reagents were purchased RNase-free and of analytical grade and were used without further purification.

### Synthesis of mRNA

mRNA was synthesized following a previously reported protocol.^6,10^ Briefly, firefly luciferase-encoding plasmid pTK305 was linearized and used as a DNA template for in vitro transcription by means of a T7 RNA polymerase kit with complete substitution of uridine by N1-methylpseudouridine. The resulting RNA was purified by LiCl/EDTA precipitation and resuspended in an SH buffer.

### Preparation of LNP-mRNA Solutions

Luciferase N1-methylpseudouridine-mRNA (10 μg) in SH buffer (120 mM NaCl, 20 mM HEPES, pH 7.4) was mixed with 7.5 μL of 1 M Na_2_SO_4_ to reach a final Na_2_SO_4_ concentration of 62.5 mM. Lipofectamine-2000 was added at various mRNA-to-lipid weight ratios (w/v) to prepare mRNA–LNP mixtures with the following amounts of LNP: 90 μL (10% mRNA), 23.3 μL (30% mRNA), 10 μL (50% mRNA), 6 μL (60% mRNA), 4.3 μL (70% mRNA), 2.5 μL (80% mRNA), 1.1 μL (90% mRNA), and 0 μL (100% mRNA). The total mixture was adjusted to a final volume of 120 μL by adding SH buffer. For normalization and quantitative Raman spectral analysis, all spectra were normalized using an internal standard of Na_2_SO_4_ at a 62.5 mM concentration.

### UV–Vis Absorption Spectrometer

To determine the appropriate excitation laser wavelength, UV–vis absorption spectra of AMP and mRNA were measured using an S-3100 (Scinco, Korea) spectrometer equipped with 1 mm path length quartz cuvettes. The spectra were collected over the 200–800 nm range.

### Deep-UV Resonance Raman

A home-built deep-UV Raman spectrometer was utilized.^11^ The fourth harmonic (266 nm) of the Nd:YAG laser was used to generate DUVRR spectra. The UV laser was focused within a Suprasil NMR tube (SP-Wilmad-LabGlass) containing about 150 μL of the solution, and a magnetic stir bar ensured continuous mixing of the sample to prevent photodegradation. The scattered light was collected in a backscattering geometry and coupled to a double monochromator optimized for UV and deep-UV spectral regions with a CCD camera (Roper Scientific) cooled with liquid nitrogen. A 100 nm slit was selected for the optimum spectral resolution and signal intensity. WinSpec32 (Roper Scientific) was used for DUVRR Spectra data collection. The laser power on the sample was approximately 5 mW. Each DUVRR spectrum represents an average of 20 accumulations, each of which was 90 s long.

### Data Treatment

Data processing, including smoothing, baseline correction, and normalization, was performed using PLS_Toolbox ver. 8.0 (Eigenvector Research, Inc., Manson, WA, USA) in MATLAB R2020b (The MathWorks Inc., Natick, MA, USA) before the principal component analysis (PCA) and 2D correlation analysis calculations. Smoothing was conducted by using Savitzky–Golay filter with a 15 nm filter width. The Whittaker filter was used for baseline correction with a Lambda value of 1000 and a *p*-value of 1 × 10^–5^. 62.5 mM of Na_2_SO_4_ (62.5 mM) was used as an internal standard. All spectra were normalized using a 977 ± 1 cm^–1^ Na_2_SO_4_ Raman band. Synchronous and asynchronous 2D correlation spectra were obtained using homemade code in MATLAB R2020b software.

## Results and Discussion

DUVRR spectroscopy was employed to examine how varying concentrations of LNPs influence the vibrational characteristics of mRNAs. To achieve a range of encapsulation, from fully encapsulated to completely free mRNA, the mRNA-to-lipid ratio was systematically changed while maintaining a constant mRNA concentration. As shown in Figure S1, both adenosine monophosphate (AMP) and mRNA exhibit strong absorption near 260 nm, corresponding to the π–π* electronic transition of the nucleobases. Therefore, an excitation wavelength of 266 nm was chosen to achieve a resonance enhancement of the Raman signal through these nucleobases. The resulting spectra were analyzed to identify vibrational bands sensitive to lipid–mRNA interactions and to establish spectral markers that can distinguish between free and encapsulated mRNA.

### Raman Spectra of Single Nucleotides

To start our analysis, we assigned the characteristic DUVRR bands found in RNAs.

mRNA is a long single-stranded molecule composed of four nucleotides: adenosine monophosphate (A), cytosine monophosphate (C), guanosine monophosphate (G), and uridine monophosphate (U). At 266 nm excitation, DUVRR spectra of mRNA are dominated by purines, A and G. Since the enhancement factor of A is greater than that of G, producing significantly stronger DUVRRS signals,^12^ adenosine monophosphate (AMP) was selected to represent the bands found in mRNA (Figure S2). The characteristic bands at 1335 and 1485 cm^–1^ correspond to the ring breathing mode of A and G.^6,12^ The bands at 1307, 1378, and 1424 cm^–1^ are influenced by both the nucleobase and the ribose ring. The band assignments of AMP based on the literature data are shown in Table S1.

### Raman Spectra of mRNA/LNP Mixture

To assess the impact of LNP encapsulation on the Raman vibrational features of mRNA, we analyzed the DUVRR spectra of samples containing 10 μg of mRNA mixed with varying volumes of added lipids (w/v): 90 μL (resulting in 10% mRNA in the LNP mixture), 23.3 μL (30% mRNA in the LNP mixture), 10 μL (50% mRNA), 6 μL (60% mRNA), 4.3 μL (70% mRNA), 2.5 μL (80% mRNA), 1.1 μL (90% mRNA), and 100% mRNA when no lipids were added. This approach allowed us to investigate how the different levels of encapsulation influence the overall spectral intensity and the presence or absence of specific vibrational bands. By comparing the spectra across various formulations, we aimed to identify distinct spectral signatures associated with free mRNA, lipid-bound mRNA, and lipid-related components.

Figure 2A shows the DUVRR spectra of the buffer, LNPs dispersed in the buffer, free mRNA without LNPs (denoted as the 100% mRNA sample), and mixtures of mRNA with LNPs. Under 266 nm excitation, LNPs exhibit a spectral profile nearly identical with that of the buffer solution. The weak spectra observed for the 10% and 30% mRNA samples can be attributed to strong turbidity and partial sedimentation, which reduce the effective optical sampling volume. Similar aggregation behavior in lipid-dominant, low-mRNA mixtures has been reported previously.^13^

**Figure 2:**
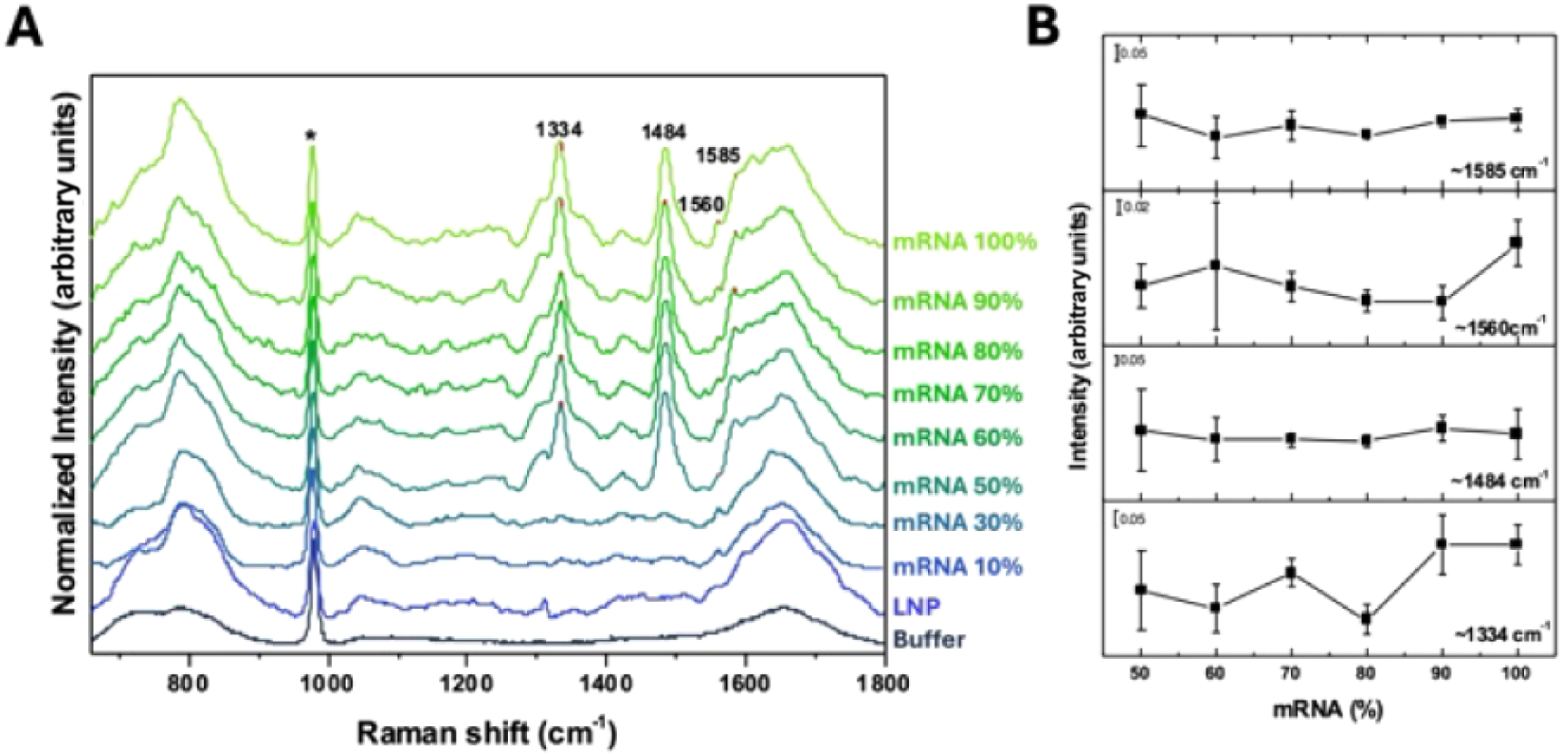
DUVRR spectra of the buffer solution, lipid nanoparticles (LNPs) in buffer, and samples prepared with 10 μg of mRNA mixed with varying volumes of LNPs, corresponding to the following mRNA-to-lipid ratios (w/v): 10%, 30%, 50%, 60%, 70%, 80%, and 90%. Free mRNA without lipid in buffer is denoted as 100% mRNA (A). The intensities of DUVRR bands near 1334, 1484, 1560, and 1585 cm^–1^ are plotted for mRNA weight ratios relative to LNPs from 50% to 100% (B). The asterisk marks indicate the internal standard Na_2_SO_4_ band.

Figure 2B shows the intensity of DUVRR bands near 1334, 1484, 1560, and 1585 cm^–1^ plotted as a function of the mRNA weight ratio relative to that of LNPs. These bands were selected based on their clear presence in the AMP spectrum (Figure S2) and were expected to respond to changes in the amount of lipid. While the bands at 1484 and 1585 cm^–1^ showed no significant variation across different mRNA:LNP ratios, the intensities of the bands at 1334 and 1560 cm^–1^ changed as the mRNA:LNP ratio increases from 80% to 100% and from 90% to 100%, respectively (*p* < 0.05).

The band at 1334 cm^–1^ mainly originates from coupled vibrations of the phosphate backbone and adenine ring, as confirmed by the DFT-calculated vibrational modes of AMP (Figure S3A). Due to electrostatic interactions between ionizable lipids and the phosphate backbone, this vibrational mode exhibits heightened sensitivity to vibrations in lipid content than other modes. Such interaction can affect the overall polarizability of mRNA, thereby resulting in the observed intensity changes.^14^ The band at 1560 cm^–1^ (Figure S3B) corresponds to ring breathing of the nucleobase. Although its intensity slightly increases with higher mRNA ratios, the large standard deviation at 60% and the *p*-value greater than 0.05 indicate that this variation is not statistically significant. This fluctuation reflects structural heterogeneity within the LNPs at this composition, where most mRNA molecules are encapsulated, but some are surrounded by less compact lipid domains compared with the fully encapsulated state.^15^

### PCA of mRNA/LNP Mixture

PCA simplifies complex data sets by reducing dimensionality while preserving essential information, thereby enhancing data visualization and interpretation. In this study, PCA was applied to explore the complex interactions between mRNA and LNPs and to identify meaningful spectral changes. The analysis revealed correlations between different components of the mRNA mixture and their corresponding spectral features, providing insights into how lipid molecules affect the spectral characteristics of mRNA. Figure 3 displays the score plots of the first two PCs and the corresponding loading vectors in the 1270–1800 cm^–1^ range. In Figure 3A, the PC1 scores show minimal variation in the 50–70% mRNA but exhibit a steady change from 80% to 100% mRNA, indicating a transition toward spectral features characteristic of free mRNA. The shift in PC1 scores from negative to positive between 80% and 90% mRNA suggests a significant change in the dominant bands. PC2 scores show a more subtle transition from negative to positive between 60% and 70% mRNA, which corresponds to spectral features indicative of mRNA–LNP interactions. Based on these transitions, the mRNA ratio range was divided into three groups: 50–60% (red), 70–80% (green), and 90–100% (blue), with corresponding spectral bands highlighted in Figure 3B-D. In the 50–60% range, the bands at 1407, 1464, 1493, 1658, 1670, and 1770 cm^–1^ were attributed to LNP-related vibrations. In the 70–80% mRNA range, the bands at 1430, 1441, and 1730 cm^–1^, which appeared negative in PC1 and positive in PC2, were also attributed to LNP molecules, particularly cholesterol, which is known to stabilize the structure of LNPs.^16^ These bands act as distinctive features of a transitional region, where partial mRNA encapsulation and lipid–mRNA interactions occur. These results suggest that LNP-related components predominantly contribute to the major PCs in the 50–80% mRNA range.

**Figure 3:**
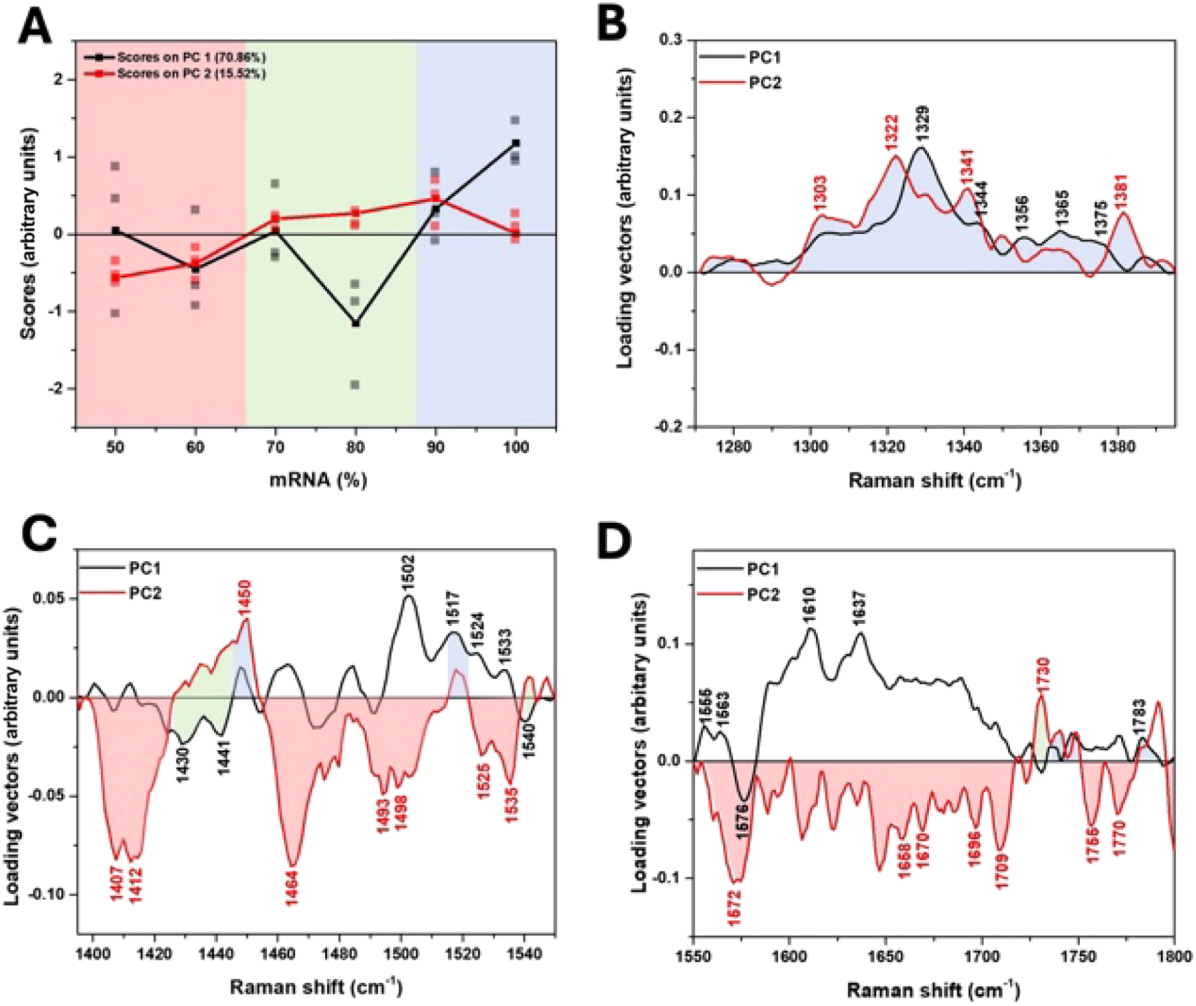
Plots of score values for the first two PCs as a function of the mRNA ratio. The black and red square marks represent the average score values, and the gray and pink square marks represent individual measurement scores (A). The corresponding loading vectors (B, C, and D) were obtained from the mRNA ratio-dependent DUVRR spectra in the regions of 1270–1395 cm^–1^, 1395–1550 cm^–1^, and 1550–1800 cm^–1^, respectively. The red area represents bands mainly contributing at 50–60% mRNA, the green area at 70–80% mRNA, and the blue area at 90–100% mRNA.

On the other hand, in the 90–100% mRNA range, bands in the 1270–1395 cm^–1^ region became prominent, all attributed to nucleotides in mRNA. Interestingly, although the overall lipid amount decreases, the influence of the PC2 bands with positive values at 1322 (G), 1341 (A and G), and 1381 cm^–1^ (G) becomes stronger at 90% mRNA. This is likely because more mRNA is exposed to the buffer, resulting in a more pronounced interaction with the remaining lipid molecules. At 100% mRNA, the PC2 influence becomes negligible and the influence of PC1 bands with positive values at 1329, 1344, and 1365 cm^–1^, corresponding to intrinsic mRNA signals, becomes dominant (Figure 3B). As the concentration of lipid decreases, the lipid-associated mRNA decreases, exposing more of the mRNA to the buffer. This transition led to a reduction in lipid–mRNA interaction bands (PC2) and a shift toward pure signals (PC1). For 100% mRNA, hydrogen bonding occurs without interference from the lipids; only the influence of the PC1 positive bands (1329, 1344, and 1365 cm^–1^) remains. The band assignments for LNP-dependent DUVRR spectra of mRNA according to the loading vectors are shown in Table S2.

### 2D-COS Analysis of mRNA/LNP Mixture

To better understand if the decrease in PC2 contribution at 100% mRNA is due to the absence of the effect of lipids, the region indicative of positive PC2, 1270–1395 cm^–1^ (Figure 3B), was analyzed using two-dimensional correlation spectroscopy (2D-COS). The 2D-COS analysis provides more information to (i) determine the number of bands within this region, (ii) assess whether the bands at 1322 and 1329 cm^–1^ represent shifts or are distinct bands, and (iii) identify the temporal relationship between the bands.

In the 1320–1340 cm^–1^ region, the synchronous 2D correlation spectrum shows a broad single autopeak (Figure 4A), whereas it was clearly resolved into two separate bands at 1322 cm^–1^ and 1329 cm^–1^ in the asynchronous 2D correlation spectrum (Figure 4B). Since no butterfly pattern in the 1320–1340 cm^–1^ region is observed in the asynchronous 2D correlation spectrum, it indicates that the feature does not result from a single band shifting position but rather from two overlapping bands. This clearly confirms that these two bands coexist as independent vibrational modes.^17^ In addition, the sequential order of intensity changes can be determined from the comparison of the signs of cross-peaks in the synchronous and asynchronous 2D correlation spectra. If the signs of cross-peaks are the same, the intensity change at *ν*_1_ occurs before *ν*_2_, while, if different, *ν*_2_ occurs before *ν*_1_. Based on the signs as shown in Table S3, the sequence of intensity changes for bands in the 1270–1395 cm^–1^ region suggests that bands with positive PC2 values (1381, 1322, and 1303 cm^–1^) change earlier than bands with positive PC1 values (1329, 1344, 1365, 1356, and 1375 cm^–1^). This supports the notion that the dominant bands at PC2, which are likely related to the lipid interactions, change first, followed by dominant bands at PC1, which are likely unrelated to the lipid interactions.

**Figure 4:**
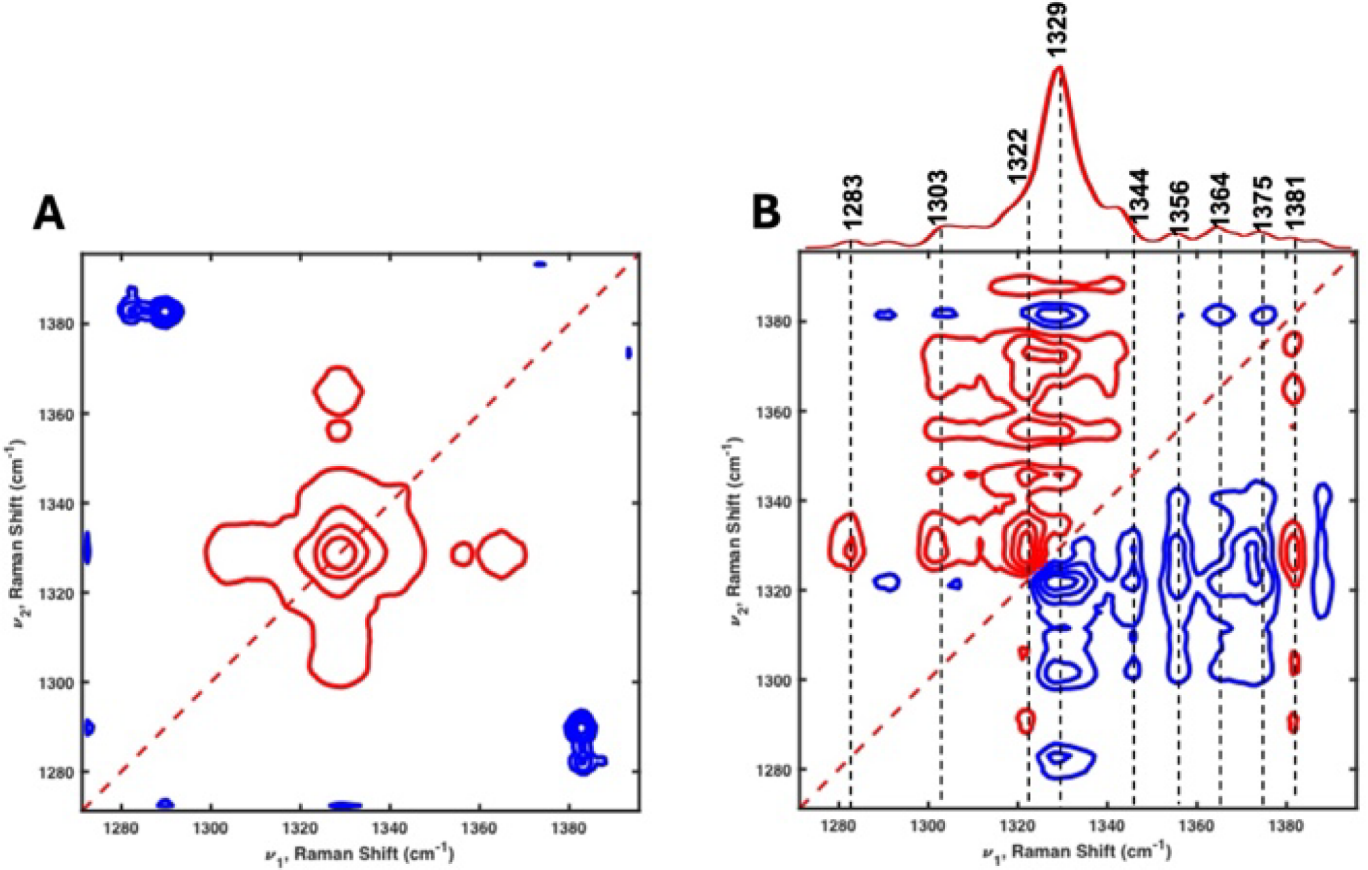
Synchronous (A) and asynchronous (B) 2D correlation DUVRR spectra of the lipid-dependent mRNA in the 1270–1395 cm^–1^ region. The power spectrum shown above the asynchronous spectrum was derived from the diagonal of the synchronous spectrum. The red and blue lines represent positive and negative cross-peaks, respectively.

As previously mentioned, the seemingly simple bands in the DUVRR spectra of mRNA mixed with lipids consist of multiple overlapping bands due to interactions between the lipids and nucleosides. Therefore, to identify reliable quantitative markers based on the lipid ratio, it is necessary to differentiate bands originating from pure mRNA and those from mRNA interacting with lipids. To extract this information by comparing pairs of spectra, we used two-trace two-dimensional correlation spectroscopy (2T2D-COS). The 2T2D-COS method is a novel method to analyze the differences between the two spectra. Although 2T2D-COS has traditionally been used as a qualitative tool to visualize spectral differences, in this study, it was employed quantitatively to assess the magnitude of the spectral variation related to lipid interactions. In 2T2D-COS, employing the DUVRR spectrum of 100% mRNA as a reference allows us to effectively separate the spectral contribution of lipid interactions, distinguishing them from bands inherent to the mRNA. This approach has the advantage of improved spectral resolution, separating overlapping bands and identifying bands affected by lipid interactions relative to those unique to the mRNA.

In 2T2D-COS, the synchronous correlation spectrum mainly provides information about simultaneous changes in spectral intensity and may not be helpful in distinguishing the differences between the two spectra. The asynchronous correlation spectrum, however, shows independent changes in the spectral intensity between two spectral variables. Therefore, in this study, we will focus on only the asynchronous correlation spectra. The sign of the cross-peak is determined by the formula: *J*(ν_1_,ν_2_) = $\frac{1}{2}$ ­[*s*(ν_1_)*r*(ν_2_) − *s*(ν_2_)*r*(ν_1_)], (*s*: sample, *r*: reference). In general, in an asynchronous 2T2D correlation spectrum, a positive cross-peak at (*ν*_1_, *ν*_2_) indicates that the *ν*_1_ component is more dominant than *ν*_2_ in the sample spectrum with respect to the reference spectrum.^7^

In the asynchronous 2T2D correlation spectra shown in Figure 5, the DUVRR spectrum of 100% mRNA is the reference spectrum, and those of 50%, 60%, 70%, 80%, and 90% mRNA are the sample spectra. Figure 5 shows the average spectrum, and similar results across three independent experiments are presented in Figure S4. The asynchronous 2T2D correlation spectra obtained from the DUVRR spectrum of 100% mRNA and those of 50% (Figure 5A) and 60% mRNA (Figure 5B) suggest that the bands at 1322 and 1329 cm^–1^ are not yet clearly separated, but the broad band at 1317–1329 cm^–1^ is more dominant than 1334 cm^–1^ in the 50% and 60% mRNA samples. In contrast, in the 70% and 80% mRNA spectra, the 1328 cm^–1^ band is more dominant than 1322 cm^–1^ in these two concentrations. For the 90% mRNA, the spectrum is almost identical to the reference spectrum of the 100% mRNA, with the sign of cross-peaks approaching zero. As a result, the asynchronous spectrum is noisier, reflecting the minimal difference between the sample and the reference. Therefore, the 2T2D-COS analysis clearly illustrates how the spectral pattern progressively evolves toward that of 100% mRNA sample as the lipid content decreases. A quantitative comparison of these spectral differences is further presented in Figure 5, which shows the correlation intensity at 1322 cm^–1^ extracted from the 1334 cm^–1^ slice.

**Figure 5:**
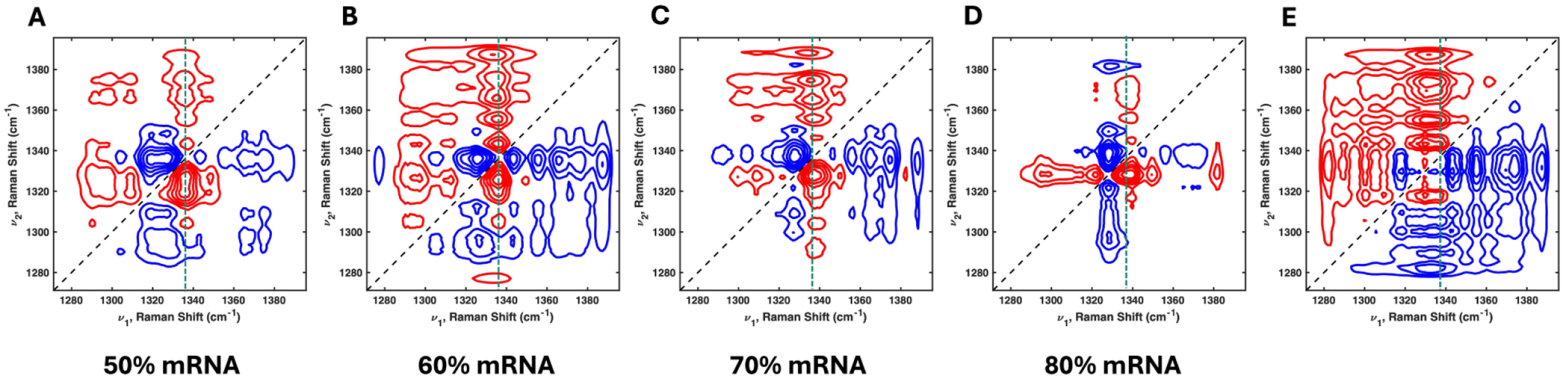
Asynchronous 2T2D correlation spectra obtained using the DUVRR spectrum of 100% mRNA as the reference spectrum and the mRNA ratios to lipid as the sample spectrum in the 1270–1395 cm^–1^ region. 50% mRNA (A), 60% mRNA (B), 70% mRNA (C), 80% mRNA (D), and 90% mRNA (E) spectra are presented. The green dashed lines indicate the 1334 cm^–1^ band.

Figure 6A displays slice spectra extracted along 1334 cm^–1^ in asynchronous 2T2D correlation spectra, showing that the 50% mRNA sample exhibits two bands near ∼1320 and ∼1326 cm^–1^. The intensity of the band at 1320 cm^–1^ is higher than that at the 1326 cm^–1^ band. As the mRNA ratio increases, the prominence of the band at 1322 cm^–1^ decreases and only the 1329 cm^–1^ band is visible at 80% mRNA. This result is consistent with the previous PCA and 2D-COS analyses and supports the conclusion that the band at 1322 cm^–1^ is associated with lipids and the band at 1329 cm^–1^ is characteristic of free mRNA. In addition, a linear decrease in the correlation intensity at 1322 cm^–1^, with an *R* ^2^ of 0.94, was observed across the sliced spectra (Figure 6B). This linearity can be explained by the fact that no lipid-related bands are present in 100% mRNA, simplifying the relationship to $J\left(1322,1334\right)=\frac{1}{2}\left[s\left(1322\right)r\left(1334\right)-s\left(1334\right)r\left(1322\right)\right]$ . Thus, this study demonstrates the potential of 2T2D-COS as a quantitative tool to investigate the distribution of lipids and mRNA and is the first application of 2T2D-COS to quantitative analysis.

**Figure 6:**
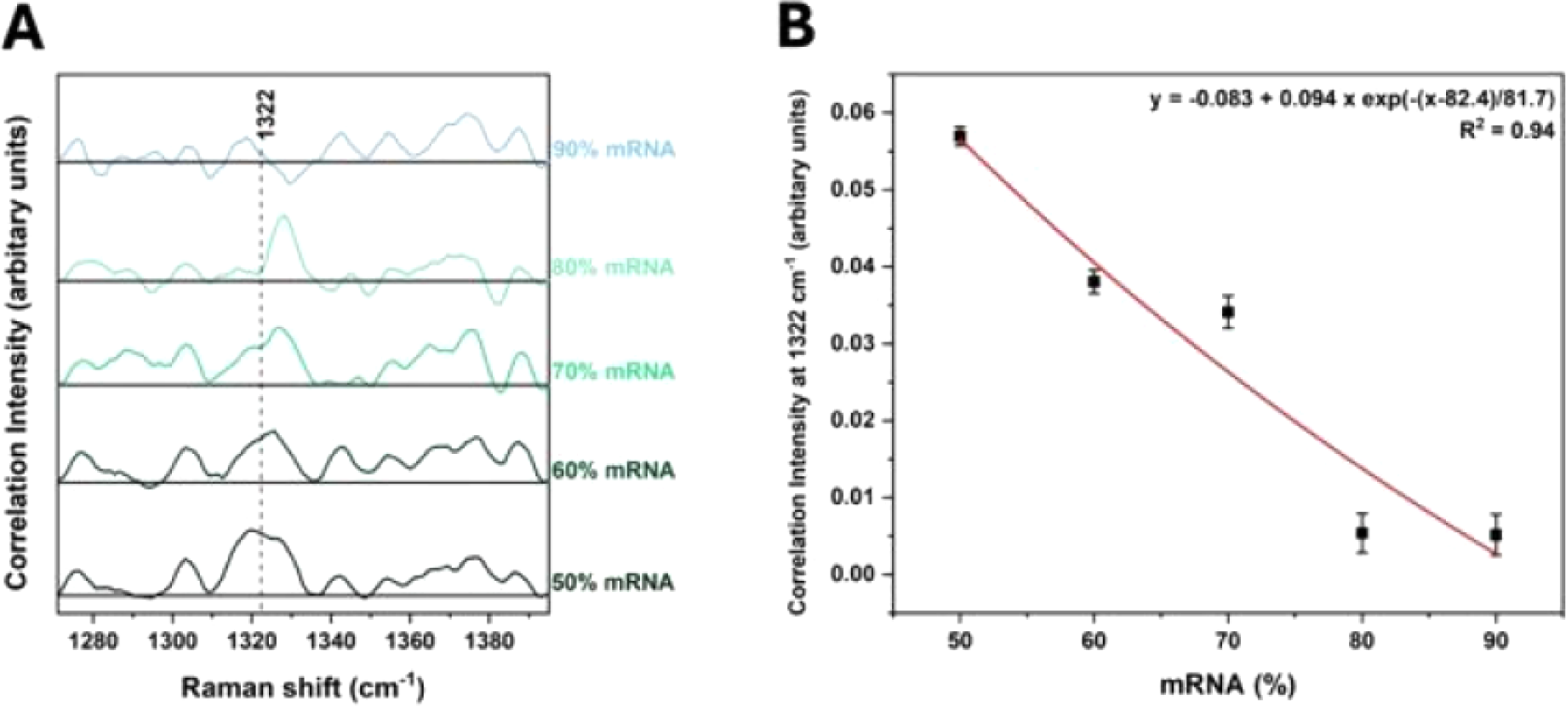
Slice spectra extracted along 1334 cm^–1^ in asynchronous 2T2D spectra are shown in Figure 5. All spectra were obtained using the asynchronous 2T2D correlation function $J\left(1334,\nu_{2}\right)=\frac{1}{2}\left[s\left(1334\right)r\left(\nu_{2}\right)-s\left(\nu_{2}\right)r\left(1334\right)\right]$ , where *s* and *r* denote the sample and reference spectra, respectively. (A) Slice spectra and (B) correlation intensities at 1322 cm^–1^ extracted from (A).

## Conclusion

This study demonstrates the use of DUVRR spectroscopy, in combination with PCA and 2T2D-COS, to analyze and quantify mRNA encapsulated within LNPs. The findings reveal that specific vibrational modes in the 1270–1800 cm^–1^ range are influenced by the interaction between mRNA and LNPs. In particular, PCA and 2D-COS results highlighted the significant spectral changes in the 1270–1395 cm^–1^ range, indicating the influence of the mRNA–LNP interactions. The analysis identified key vibrational markers at 1322 and 1329 cm^–1^, corresponding to LNP-bound mRNA and free mRNA, respectively. 2T2D correlation spectra further revealed a linear change in the band at 1322 cm^–1^ with increasing mRNA ratios, underscoring its potential as a quantifiable marker for mRNA encapsulation. Notably, the correlation intensity at 1322 cm^–1^, extracted from the 1334 cm^–1^ slice in the asynchronous 2T2D correlation spectra, showed a quantitative trend. High values (∼0.035 a.u.) indicate fully encapsulated mRNA, low values (∼0.01 a.u.) reflect unencapsulated mRNA, and intermediate values represent partially encapsulated mRNA. The combined use of DUVRR spectroscopy, PCA, 2D-COS, and 2T2D-COS establishes a label-free and nondestructive analytical platform for elucidating and quantifying mRNA encapsulation within LNPs. In addition to providing molecular-level insight into lipid–mRNA interactions, this integrated approach enables rapid formulation screening and quality control of mRNA therapeutics, where encapsulation efficiency critically influences delivery and expression performance. Furthermore, the identification of quantifiable spectral markers opens opportunities for the in situ and high-throughput monitoring of mRNA–LNP formulations during production or storage. Overall, this study provides a spectroscopic framework that connects fundamental molecular characterization with a practical assessment of therapeutic performance.

## Supplementary Material



## Notes

UV–vis absorption spectra, DUVRR spectra of AMP, AMP Raman bands assignment table, DFT-calculated AMP vibrational modes, band assignments table for lipid-dependent DUVRR spectra of mRNA, 2T2D asynchronous spectra, and correlation pattern table (PDF)

## Notes

The manuscript was written through contributions of all authors. All authors have given approval to the final version of the manuscript
