# Supplementary material for: Encapsulation of mRNA in Therapeutics Like Lipid Nanoparticles Probed by Deep-UV Resonance Raman Spectroscopy
Source: Anal Chem. 2026 Jan 15;98(5):3523–30. doi: 10.1021/acs.analchem.5c04246 (PMC12903059; doi:10.1021/acs.analchem.5c04246)
Supplement: Supplementary file 1 [file ac5c04246_si_001.pdf]

Supporting Information

Encapsulation of mRNA in Therapeutics Like lipid Nanoparticles Probed by Deep-UV Resonance Raman Spectroscopy

Sila Jin[1,2], Sergei V. Reverdatto[1,3], Vladimir V. Ermolenkov[3], Alexander Shekhtman[1,3], Young Mee Jung[2,4,*], and Igor K. Lednev[1,5,*]

[1]Department of Chemistry, University at Albany, SUNY, 1400 Washington Avenue, Albany, New York 12222, United States

[2]Kangwon Radiation Convergence Research Support Center, Kangwon National University, Chuncheon 24341, Republic of Korea

[3]The RNA Institute, College of Arts and Science, University at Albany, SUNY, 1400 Washington Avenue, Albany, New York 12222, United States

[4]Department of Chemistry, Institute for Molecular Science and Fusion Technology, Kangwon National University, Chuncheon 24341, Republic of Korea

[5]Center for Biophotonic Technologhand Artificial Intelligence (CeBAI), University at Albany, SUNY, 1400 Washington Avenu, Albany, New York 12222, United States

E-mail: ymjung@kangwon.ac.kr (Y.M. Jung); ilednev@albany.edu (I.K. Lednev)

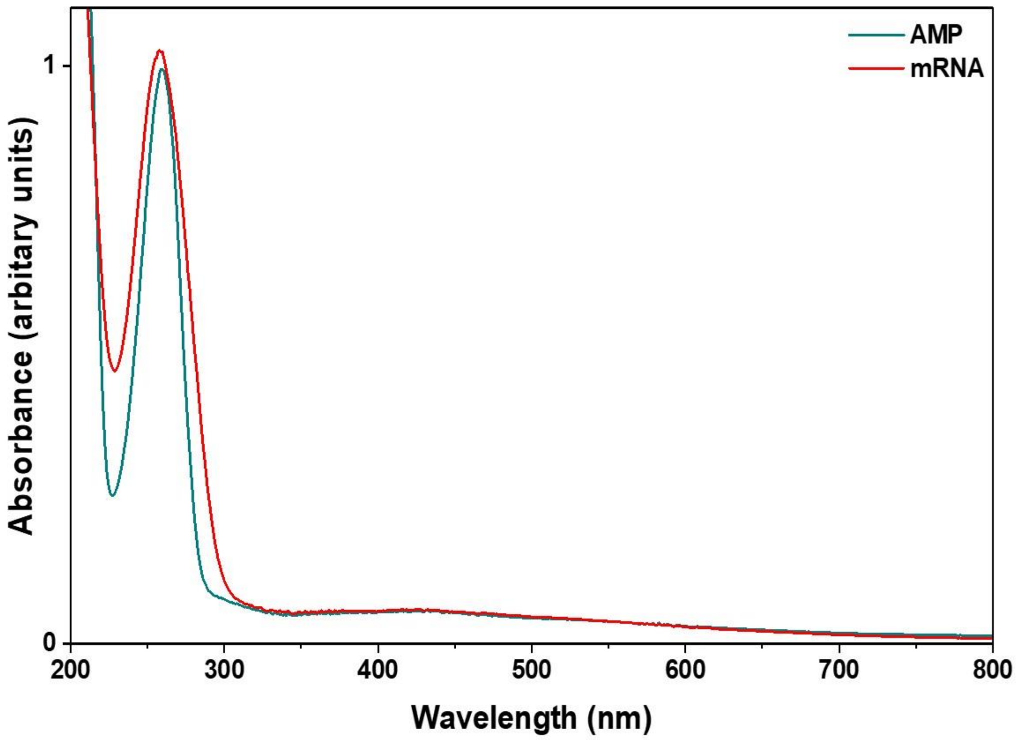

Figure S1. UV-Vis absorption spectra of adenosine monophosphate (AMP) and mRNA.

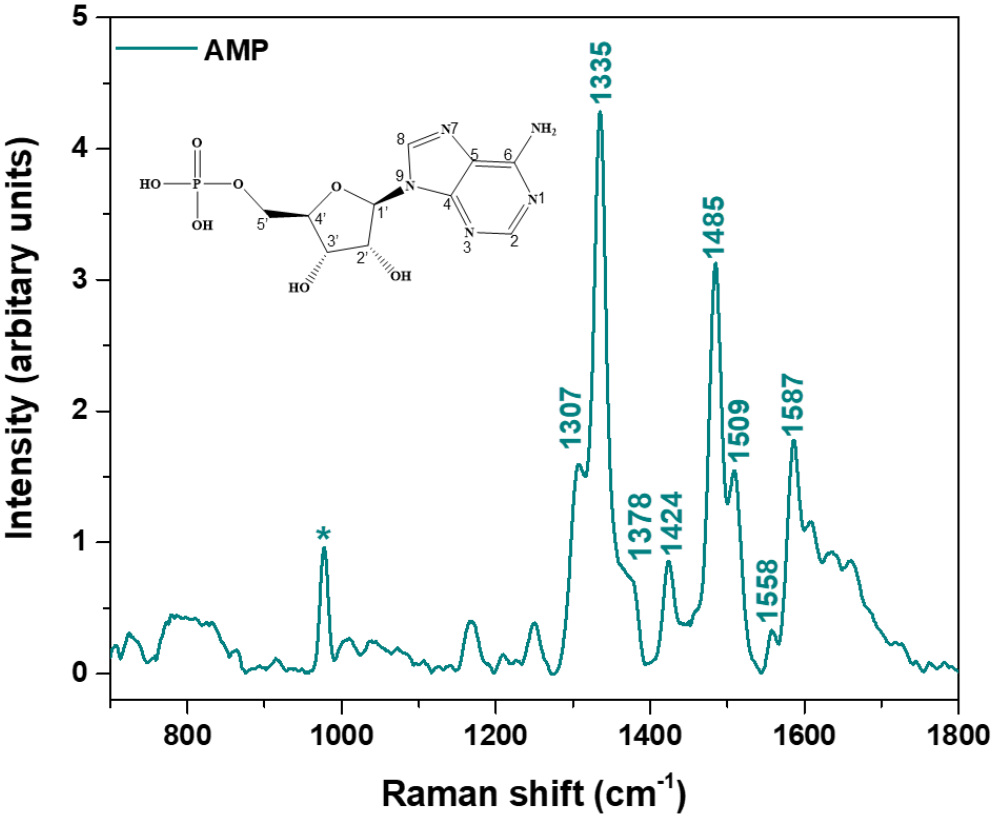

Figure S2. DUVRR spectrum of AMP. The asterisk mark indicates the internal standard Na_2SO_4 band.

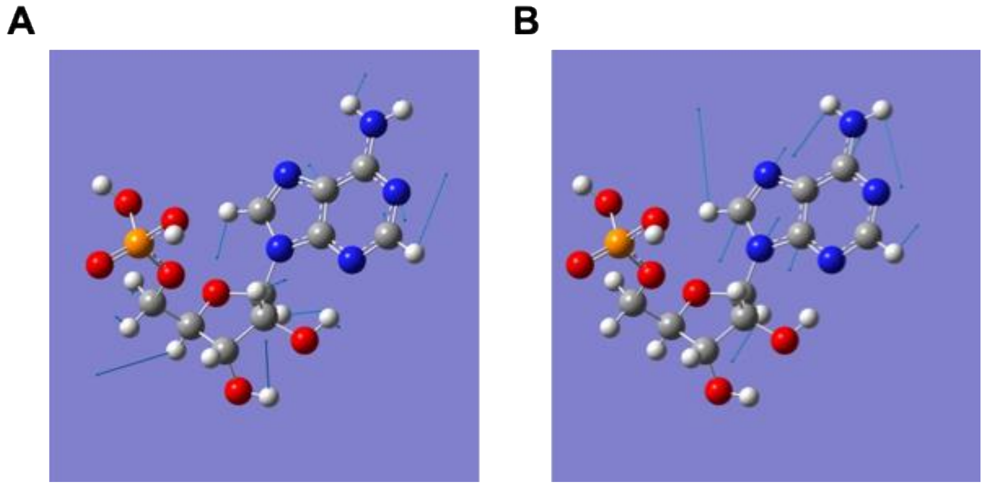

Figure S3. DFT-calculated Raman vibrational modes of AMP at 1335 cm^{-1} (A) and 1560 cm^{-1} (B). DFT calculation was performed at the B3LYP/6-311G level using *Gaussian 09*.

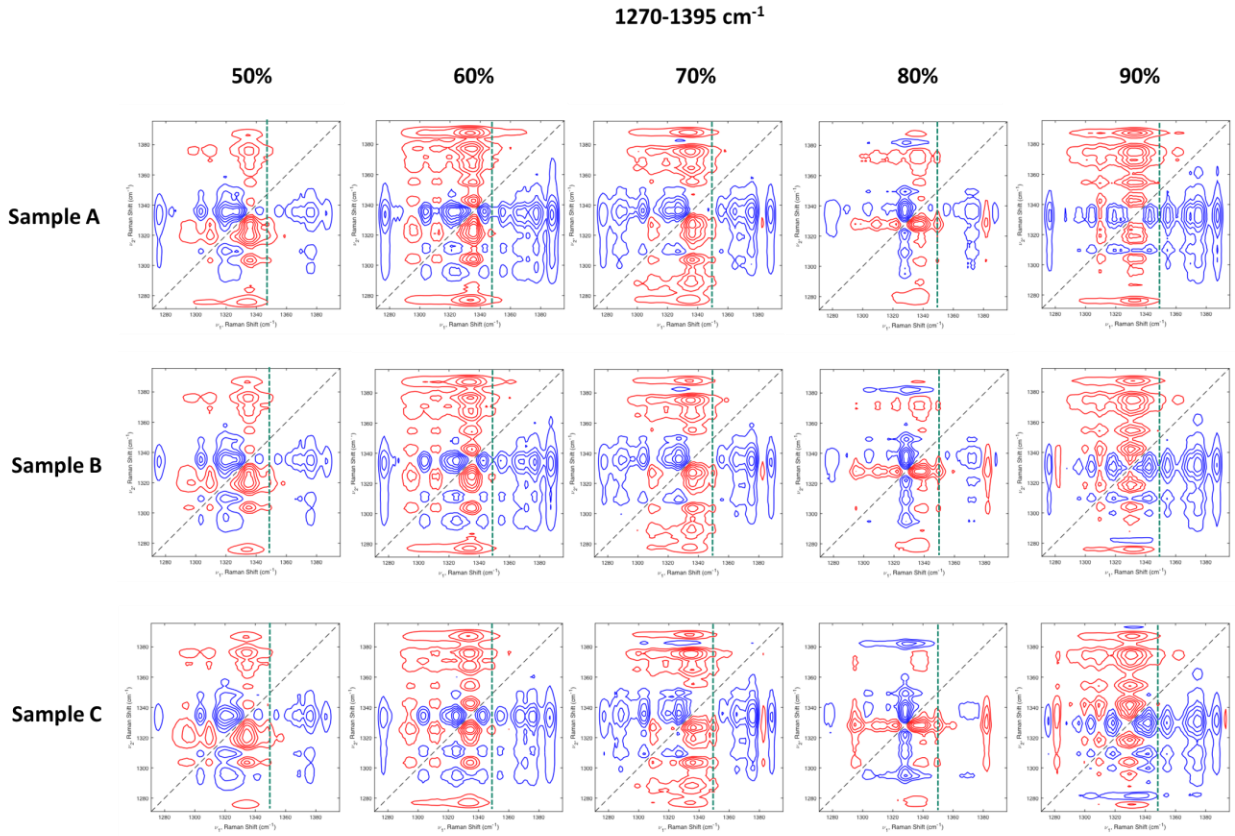

Figure S4. Individual 2T2D asynchronous spectra of LNP-dependent mRNA in the 1270-1395 cm^{-1} region. Row: Sample A, B, and C. Column: mRNA weight ratio relative to LNPs; 50, 60, 70, 80, 90, and 100%.

Table S1. Assignment of AMP Raman bands.

Raman shift (cm^{-1})	Assignments
1307	A (C-N) and (C=N) stretching[1, 2]
1335	N1C5 stretching + C8N7 stretching[1, 3]
1378	C2H + C8H bending[1]
1424	Ribose ring deformation[4]
1485	A (N1C2 stretching +N3C2 stretching)
	/ G (N1C2 stretching + N1C6 stretching)[3]
1509	A, pyrimidine stretching[5]
1558	Adenosine[6]
1587	A, G (N3C4-C4C5 stretching)[3]

Table S2. Band assignments for lipid-dependent DUVRR spectra of mRNA according to the loading vectors.

Raman shift (cm^{-1})	Assignments
1303	A (CH bending)[7]
1322	G[8]
1329	A, G (CH bending)[9]
1341	A, G[10]
1381	C-N stretching pyrimidine of guanosine[11]
1407	Lipid CH_2 bending[12]
1412	G (N7=C8-H) bending[1]
1430	Cholesterol CH_2 bending[12]
1441	Cholesterol CH_2/CH_3 scissoring[12]
1450	U (N1-C6, N1-C2 stretching, H7-N1-C6, C5C6N1 bending)[13]
1464	Lipid CH_2-CH_3 bending[12], A(5' CH_2 scissoring)[14]
1493	Lipid COO stretching[15]
1498	CH_2 scissoring of cytidine[16]
1502	A (C6–N10), C2–N3, (C6–N1) stretching, C2–H bending[17]
1517	C=C[18]
1524	C=C[18]
1533	C (N3-C4, N1-C2 stretching), G (C4-C5, C4-N9 stretching)[3]
1555	Adenosine[6]
1563	Ring stretching pyrimidine of cytidine[11]
1572	A, G (Ring Stretching pyrimidine)[11]

1610	A, C (NH_2 scissoring)[8]
1623	Adenosine[6]
1637	G (NH_2 scissoring)[19, 20]
1646	Pseudoridine (C=O stretching)[21]
1658	Lipid C=C stretching[12]
1670	Cholesterol C=C stretching[22, 23]
1696	G (C=O stretching)[19]
1709	G (C=O stretching)[24]
1730	U (C=O stretching)[13]
1755	U (C=O stretching)[13]
1770	Lipid C=O stretching[25]

Table S3. Correlation patterns were observed in the 2D correlation DUVRR spectra of the 1270 – 1395 cm^{-1} region of LNP-dependent mRNA.

**Raman shift (cm^{-1}) (ν_1, ν_2)**	**Sign (syn, asyn)**	**Assignments**
(1322, 1381)	(+, -)	(PC2, PC2)
(1303, 1322)	(+, -)	(PC 2, PC2)
(1303, 1329)	(+, +)	(PC2, PC1)
(1329, 1344)	(+, +)	(PC1, PC1)
(1344, 1365)	(+, +)	(PC1, PC1)
(1356, 1365)	(+, -)	(PC1, PC1)
(1356, 1375)	(+, +)	(PC1, PC1)

References

(1) Mathlouthi, M.; Seuvre, A.-M.; Koenig, J. L. F.t.-i.r. and laser-Raman spectra of adenine and adenosine. *Carbohydrate Research* **1984**, *131* (1), 1-15. DOI: https://doi.org/10.1016/0008-6215(84)85398-7.

(2) Oldenburg, S.; Averitt, R.; Westcott, S.; Halas, N. Nanoengineering of optical resonances. *Chem. Phys. Lett.* **1998**, *288* (2-4), 243-247.

(3) Otto, C.; van den Tweel, T. J. J.; de Mul, F. F. M.; Greve, J. Surface-enhanced Raman spectroscopy of DNA bases. *J. Raman Spectrosc.* **1986**, *17* (3), 289-298. DOI: https://doi.org/10.1002/jrs.1250170311.

(4) Ebenezar, I. J. D.; Ramalingam, S.; Raja, C. R.; Prabakar, P. J. Vibrational spectroscopic [IR and raman] analysis and computational investigation [NMR, UV-Visible, MEP and kubo gap] on L-Valinium picrate. *J. Nanotechnol. Adv. Mater* **2014**, *2* (1), 11-25.

(5) Masetti, M.; Xie, H.-n.; Krpetić, Ž.; Recanatini, M.; Alvarez-Puebla, R. A.; Guerrini, L. Revealing DNA Interactions with Exogenous Agents by Surface-Enhanced Raman Scattering. *J. Am. Chem. Soc.* **2015**, *137* (1), 469-476. DOI: 10.1021/ja511398w.

(6) Tripon, C.; Muntean, C. M.; Bratu, I.; Nalpantidis, K.; Deckert, V. (Sub)picosecond processes in DNA and RNA constituents: a Raman spectroscopic assessment. *Polymer Bulletin* **2017**, *74* (10), 4087-4100. DOI: 10.1007/s00289-017-1938-x.

(7) Huser, T.; Orme, C. A.; Hollars, C. W.; Corzett, M. H.; Balhorn, R. Raman spectroscopy of DNA packaging in individual human sperm cells distinguishes normal from abnormal cells. *Journal of Biophotonics* **2009**, *2* (5), 322-332. DOI: https://doi.org/10.1002/jbio.200910012.

(8) Ruiz-Chica, A. J.; Medina, M. A.; Sánchez-Jiménez, F.; Ramírez, F. J. Characterization by Raman spectroscopy of conformational changes on guanine–cytosine and adenine–thymine oligonucleotides induced by aminooxy analogues of spermidine. *J. Raman Spectrosc.* **2004**, *35* (2), 93-100. DOI: https://doi.org/10.1002/jrs.1107.

(9) Sacco, A.; Barzan, G.; Matić, S.; Giovannozzi, A. M.; Rossi, A. M.; D'Errico, C.; Vallino, M.; Ciuffo, M.; Noris, E.; Portesi, C. Raman-dielectrophoresis goes viral: towards a rapid and label-free platform for plant virus characterization. *Frontiers in Microbiology* **2023**, *14*, Original Research. DOI: 10.3389/fmicb.2023.1292461.

(10) Ravi, P.; Singh, S. P.; Kang, J. W.; Tran, S.; Dasari, R. R.; So, P. T. C.; Liepmann, D.; Katti, K.; Katti, D.; Renugopalakrishnan, V.; et al. Spectrochemical Probing of MicroRNA Duplex Using Spontaneous Raman Spectroscopy for Biosensing Applications. *Anal. Chem.* **2020**, *92* (21), 14423-14431. DOI: 10.1021/acs.analchem.0c02401.

(11) Jang, N.-H. The Coordination Chemistry of DNA Nucleosides on Gold Nanoparticles as a Probe by SERS. *Bulletin of the Korean Chemical Society* **2002**, *23* (12), 1790-1800. DOI: 10.5012/BKCS.2002.23.12.1790.

(12) Czamara, K.; Majzner, K.; Pacia, M. Z.; Kochan, K.; Kaczor, A.; Baranska, M. Raman spectroscopy of lipids: a review. *J. Raman Spectrosc.* **2015**, *46* (1), 4-20. DOI: https://doi.org/10.1002/jrs.4607.

(13) Sun, S.; Brown, A. Simulation of the Resonance Raman Spectrum for Uracil. *J. Phys. Chem. A* **2014**, *118* (39), 9228-9238. DOI: 10.1021/jp503099m.

(14) Thomas, G. J.; Benevides, J. M.; Overman, S. A.; Ueda, T.; Ushizawa, K.; Saitoh, M.; Tsuboi, M. Polarized Raman spectra of oriented fibers of A DNA and B DNA: anisotropic and isotropic local Raman tensors of base and backbone vibrations. *Biophysical Journal* **1995**, *68* (3), 1073-1088. DOI: https://doi.org/10.1016/S0006-3495(95)80282-1.

(15) Carter, E. A.; Edwards, H. G. *Biological applications of Raman spectroscopy*; Marcel Dekker, Inc.: New York, NY, USA, 2001.

(16) Pezzotti, G. Raman spectroscopy in cell biology and microbiology. *J. Raman Spectrosc.* **2021**, *52* (12), 2348-2443. DOI: https://doi.org/10.1002/jrs.6204.

(17) Merk, V.; Speiser, E.; Werncke, W.; Esser, N.; Kneipp, J. pH-Dependent Flavin Adenine Dinucleotide and Nicotinamide Adenine Dinucleotide Ultraviolet Resonance Raman (UVRR) Spectra at Intracellular Concentration. *Applied Spectroscopy* **2021**, *75* (8), 994-1002. DOI: 10.1177/00037028211025575 (acccessed 2024/08/16).

(18) Movasaghi, Z.; Rehman, S.; Rehman, I. U. Raman Spectroscopy of Biological Tissues. *Applied Spectroscopy Reviews* **2007**, *42* (5), 493-541. DOI: 10.1080/05704920701551530.

(19) Yu-Bo, L.; Yao-Yong, M.; Hao-Dong, L.; Ying, W. Raman spectroscopic study of G—A mismatches. *Chinese Physics* **2007**, *16* (11), 3332.

(20) Toyama, A.; Hamuara, M.; Takeuchi, H. Correlation between vibrational frequencies and hydrogen bonding states of the guanine ring studied by UV resonance Raman spectroscopy of 2′-

deoxy-3′,5′-bis(triisopropylsilyl)guanosine dissolved in various solvents. *Journal of Molecular Structure* **1996**, *379* (1), 99-108. DOI: https://doi.org/10.1016/0022-2860(95)09159-9.
(21) Morla-Folch, J.; Xie, H.-n.; Alvarez-Puebla, R. A.; Guerrini, L. Fast Optical Chemical and Structural Classification of RNA. *ACS Nano* **2016**, *10* (2), 2834-2842. DOI: 10.1021/acsnano.5b07966.
(22) Hanlon, E.; Manoharan, R.; Koo, T. W.; Shafer, K.; Motz, J.; Fitzmaurice, M.; Kramer, J.; Itzkan, I.; Dasari, R.; Feld, M. Prospects for in vivo Raman spectroscopy. *Physics in Medicine & Biology* **2000**, *45* (2), R1.
(23) Simeral, M. L.; Demers, S. M. E.; Sheth, K.; Hafner, J. H. A Raman spectral marker for the iso-octyl chain structure of cholesterol. *Analytical Science Advances* **2024**, *5* (1-2), 2300057. DOI: https://doi.org/10.1002/ansa.202300057.
(24) Baumruk, V.; Gouyette, C.; Huynh-Dinh, T.; Sun, J.-S.; Ghomi, M. Comparison between CUUG and UUCG tetraloops: thermodynamic stability and structural features analyzed by UV absorption and vibrational spectroscopy. *Nucleic Acids Research* **2001**, *29* (19), 4089-4096. DOI: 10.1093/nar/29.19.4089 (acccessed 8/15/2024).
(25) Wei, X.; Jiji, R. D.; Zare, A.; Lada, B.; Li, X.; Greenlief, C. M. Deep-UV resonance Raman spectroscopy of hydrated and dehydrated model α-helical transmembrane peptides in liposomes. *J. Raman Spectrosc.* **2022**, *53* (1), 58-68. DOI: https://doi.org/10.1002/jrs.6252.
